# Role of transforming growth factor-beta 1 and connective tissue growth factor levels in coronavirus disease-2019-related lung Injury: a prospective, observational, cohort study

**DOI:** 10.1590/0037-8682-0615-2021

**Published:** 2022-07-25

**Authors:** Esra Laloglu, Handan Alay

**Affiliations:** 1 Ataturk University, Faculty of Medicine, Department of Medical Biochemistry, Erzurum, Turkey.; 2 Ataturk University, Faculty of Medicine, Department of Infectious Diseases and Clinical Microbiology, Erzurum, Turkey.

**Keywords:** COVID-19, CTGF, Lung injury, SARS-COV-2, TGF-β1

## Abstract

**Background:**

Coronavirus disease-2019 (COVID-19) results in acute lung injury. This study examined the usefulness of serum transforming growth factor-beta 1 (TGF-β1) and connective tissue growth factor (CTGF) levels in predicting disease severity in COVID-19 patients with pulmonary involvement.

**Methods:**

Fifty patients with confirmed COVID-19 and pulmonary involvement between September 2020, and February 2021 (Group 1) and 45 healthy controls (Group 2) were classified into three subgroups based on clinical severity: moderate, severe, and critical pneumonia. Serum TGF-β1 and CTGF concentrations were measured on days 1 and 7 of admission in Group 1 using an enzyme-linked immunosorbent assay. These concentrations were also measured in control cases. The mean serum TGF-β1 and CTGF levels were then compared among COVID-19 patients, based on clinical severity.

**Results:**

Significantly higher mean serum TGF-β1 and CTGF levels were observed on both days in Group 1 than in the control group. The mean serum TGF-β1 and CTGF levels on day 7 were also significantly higher than those on day 1 in Group 1. The critical patient group had the highest serum TGF-β1 and CTGF levels on both days, and the difference between this group and the moderate and severe pneumonia groups was significant. Cutoff values of 5.36 ng/mL for TGF-β1 and 626.2 pg/mL for CTGF emerged as predictors of COVID-19 with pulmonary involvement in receiver-operating characteristic curve analysis.

**Conclusions:**

TGF-β1 and CTGF are potential markers that can distinguish COVID-19 patients with pulmonary involvement and indicate disease severity. These findings may be useful for initiating treatment for early-stage COVID-19.

## INTRODUCTION

Respiratory system findings associated with coronavirus disease-2019 (COVID-19) infection derived from SARS-COV-2 virus may emerge with differing clinical severities. Advanced age, immunosuppression, cardiovascular diseases, hypertension, chronic pulmonary and renal diseases, liver diseases, malignancies, and severe obesity constitute risk factors for severe/critical COVID-19^1^.

Our knowledge of the histopathological changes in the lungs during COVID-19 infection is still limited. Changes occurring in the early stage of the disease are based on alterations in the lung tissues of patients operated on due to pulmonary tumors, and diagnosed immediately after surgery^2^, while late-stage findings rely on autopsy data^3^. Serous and/or fibrinous exudates and hyaline membranes in the alveoli, inflammatory cell infiltration, foci of fibroblastic proliferation, edema in the alveolar wall, hyaline thrombi in the vessels, and interstitial thickening accompanied by fibrosis are observed, and all signs of widespread alveolar damage are present in patients infected with SARS-CoV-2^2^ ^,^ ^3^.

The World Health Organization’s clinical classification considers COVID-19 under the headings of mild signs, pneumonia or severe pneumonia, acute respiratory distress syndrome (ARDS), severe sepsis, and septic shock^4^.

SARS-CoV-2 enters the upper respiratory tract through angiotensin-converting enzyme 2 (ACE2) receptors on the surface of the pulmonary epithelium. The virus infects pulmonary alveolar type II cells during their migration to the lower respiratory tract^5^ ^,^ ^6^.

Interstitial edema and hyaline membrane formation develop in the acute phase as a result of widespread alveolar damage in COVID-19 patients, while septal fibrosis and proliferation in fibroblasts develop in the chronic remodeling phase^7^. Whether patients will require long-term pulmonary rehabilitation after COVID-19 infection by causing chronic lung damage, is currently unclear^8^ ^,^ ^9^.

The excessive cytokine production seen in COVID-19 is associated lung injury development^10^ ^,^ ^11^. Cytokines, released as a result of macrophage , cause massive cell breakdown^12^. This is followed by a fibrotic phase involving growth factors, including connective tissue growth factor (CTGF) and transforming growth factor-beta1 (TGF-β1), in which repair mechanisms become operative^13^. TGF-β has three isoforms, TGF-β1, TGF-β2, and TGF-β3^14^. TGF-β1, in particular, leads to pulmonary fibrosis and the overproduction of extracellular matrix by stimulating the differentiation of fibroblasts into myofibroblasts^15^ ^-^ ^18^. CTGF is a protein whose expression is increased by TGF-β1, which is involved in fibroblast growth and extracellular matrix synthesis in the early period^19^ ^,^ ^20^. 

This study aimed to investigate the mechanism of lung damage development in COVID-19 patients and to determine the association between serum TGF-β1 and CTGF levels and disease severity to contribute to the treatment of severe or chronic cases.

## METHODS

### Study design and participants

Ethical approval for this study was granted by the Republic of Turkish Ministry of Health COVID-19 Scientific Research Assessment Commission and Ethical Committee at Ataturk University Medical Faculty (No:B.30.2. Ata.0.01.00/209). Fifty patients had visited the Infectious Diseases and Clinical Microbiology Department between September 2020 and February 2021 due to symptoms including cough, sore throat, fever, muscle/joint pains, nasal discharge, shortness of breath, fatigue, and sudden loss of taste or smell, and diagnosed with COVID-19 on the basis of radiological and laboratory tests and physical examination, were enrolled as Group 1. Varying levels of pulmonary involvement were observed in these patients by radiological findings. All patients were hospitalized during treatment. On the seventh day of follow-up in the moderate pneumonia group, improvement was observed in initial symptoms, and intensive care was not required. Only two members in the severe pneumonia group required admission to the intensive care unit. Mortality occurred in four patients in the critical group. Forty-five healthy individuals were enrolled in Group 2 (control group). The patient underwent provided physical examinations and laboratory test results. All members of both groups were given detailed information concerning the research, and were enrolled following the receipt of written consent forms. Individuals with cancer, high blood pressure, cardiovascular disease, diabetes mellitus, chronic renal and liver diseases, and acute or chronic inflammatory diseases were excluded.

### Definitions and diagnosis

**Group 1:** COVID-19 was confirmed by means of a SARS-CoV-2 qPCR Detection Kit (Bio-Speedy Bioeksen) employed for the detection of the SARS-CoV-2 virus. Nucleic acid isolates were obtained from the oropharyngeal and nasopharyngeal swab specimens. The diagnosis of COVID-19 was based on the interim guidelines published by the World Health Organization^4^. Patients with COVID-19 pneumonia were then classified into three subgroups based on the clinical severity during hospital admission: moderate, severe, and critical categories requiring intensive care. Clinical evaluation relied on thoracic computed tomography and laboratory tests.

Cases exhibiting findings including high fever, sore throat, muscle-joint ache, dry cough, and findings of moderate pneumonia on thoracic tomography were classified as moderate pneumonia. 

Cases with findings including high fever, sore throat, muscle-joint ache, dry cough, tachypnea **(**>30/min), severe respiratory distress or SpO_2_ levels < 90%, and bilateral diffuse pneumonia on thoracic tomography were classified as severe pneumonia.

Critical illness was defined based on criteria applicable in cases of severe respiratory distress, sepsis, septic shock, or other conditions that would necessitate mechanical ventilation or vasopressor therapy.

**Group 2** comprised healthy individuals. All members of Group 2 were RT-PCR-negative upon enrolment in the study.

### Blood specimens

Blood specimens were collected from all patients on the first day for routine biochemistry tests, prior to commencement of any drug therapy and on the seventh day after the start of treatment^21^ ^,^ ^22^. Blood specimens were allowed to clot for 30 min and then centrifuged at +4°C for 15 min. The resulting serum specimens were stored at -80°C until TGF-β and CTGF analyses.

### Analyte assay techniques

TGF-β1 and CTGF values were determined using the ELISA method with a Human TGF-β1 ELISA kit (Elabscience, Human TGF-β1: cat. E-EL-H0110, Texas, USA) and a human CTGF ELISA kit (Elabscience, Human CTGF: E-EL-H0828, Texas, USA) in accordance with the manufacturer’s recommendations. The measurement ranges for TGF-β1 and CTGF were 31.25-2000 pg/mL and 62.5-4000 pg/mL, respectively. For TGF-β1, the kit exhibited an intra-assay coefficient of variation (CV) of 5.4% and an inter-assay CV of 4.8%. For CTGF, the intra-assay CV was 5.1% and the inter-assay CV was 4.9%.

 Serum C-reactive protein (CRP, mg/L) and lactate dehydrogenase (LDH, U/L) levels investigated during routine tests were measured on a Beckman Coulter AU5821 device present in the biochemistry laboratory, ferritin (ng/mL) and troponin-I (ng/L) levels were measured on a Beckman Coulter DxI800 device, and procalcitonin levels (ng/mL) on a Roche Cobas 6000 device, using commercial kits. The erythrocyte sedimentation rate (ESR) was measured on a StaRRsed device using the Westergren method, and the results were presented in mm/h. White blood cell (WBC), lymphocyte, and neutrophil counts were measured using a Sysmex XN-9000 device (Japan) and expressed as cells/µL. D-dimer levels (ng/mL) were determined using a Radiometer AQT90 Flex device.

### Statistical analysis

Data were recorded and analyzed using SPSS software for Windows (version 20.0; SPSS Inc., Chicago, IL, USA). Categorical variables were presented as numbers and percentages. Numerical variables are presented as mean ± standard deviation. Visual (histogram) and analytical methods (Kolmogorov-Smirnov or Shapiro-Wilk tests) were used to evaluate the normality of the distribution of variables. The chi-square test was applied to compare categorical variables, and the *t*-test or one-way analysis of variance, as appropriate, was used to compare continuous variables. The significance of differences between groups was assessed using the post-hoc Tukey test. Pearson’s correlation coefficient was used for linear correlation analysis. The receiver-operating characteristic curve (ROC) method, which indicates the predictive power of a specific method, was employed to determine TGF-β1 and the sensitivity, specificity, area under the curve (AUC), and cutoff values. Statistical significance was set at *P* < 0.05.

## RESULTS

Group 1 comprised 24 (48%) women and 26 (52%) men, while the control group comprised 21 (46.7%) women and 24 (53.3%) men. The mean ages were 47.02 ± 15.22 and 51.69 ± 16.17 years in Group 1 and the control group, respectively. No significant age or sex differences were observed between the groups (*P* = 0.89 and *P* = 0.16, respectively). When COVID-19 patients were classified in terms of clinical characteristics, moderate pneumonia was present in 20 (40%), severe pneumonia in 15 (30%), and critical pneumonia in 15 (30%). The patients' symptoms on hospital admission and clinical outcomes are shown in Table 1. Radiological examination of the patients revealed a ground glass appearance in the pulmonary parenchyma, ground glass together with consolidation, septal thickening, fibrosis, air bronchograms, reverse halo sign, nodule, and pleural effusion (Supplementary Table 1).

**TABLE 1:** Patients' symptoms at hospital admission and clinical outcomes.

General data	Overall (n = 50)
Onset-inpatient interval (n)	50 (100%)
**Signs and symptoms on admission**	
High fever	50 (100%)
Dry cough	50 (100%)
Expectoration	22 (44%)
Dyspnea	32 (64%)
Muscle ache	27 (54%)
Headache	15 (30%)
Sore throat	18 (36%)
Chest tightness	15 (30%)
Nausea and vomiting	5 (10%)
**Treatment**	n = 50
Oxygen therapy	30 (60%)
Intensive care unit	15 (30%)
Antibiotic treatment	30 (60%)
Antiviral treatment	31 (62%)
Glucocorticoids	28 (56%)
**Clinical outcome**	
Length of hospitalization (days)	
Median (min-max)	9 (4-30)
Died	4 (8%)

**SUPPLEMENTARY FIGURE 1:**
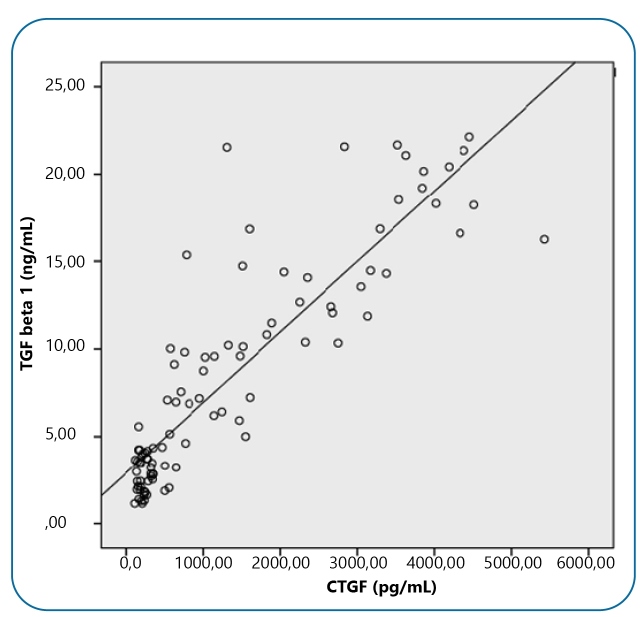
Correlation chart between serum TGF-β1 and CTGF levels on days 1 and 7 of admission in Group 1.

TGF-β1 and CTGF levels and routine laboratory parameters of the groups are shown in Table 2. Routine test results on day 1, such as LDH, D-dimer, troponin-I, ESR, CRP, and ferritin levels, were significantly higher in the patient group than in the control group (*P* < 0.05), while WBC, lymphocyte, and neutrophil counts were lower than those in the control group (*P* < 0.05). Routine laboratory test results on day 7 after medical treatment were similar to those of the control group (*P* > 0.05). However, the serum TGF-β1 and CTGF levels on day 1 and 7 were significantly higher than those in the control group (*P* < 0.05). Moreover, serum TGF-β1 and CTGF levels on day 7 were significantly higher than those on day 1 (*P* < 0.001). A strong, significant, positive correlation was observed between serum TGF-β1 and CTGF levels (r=0.884, *P* < 0.001) (Supplementary Figure 1).

**TABLE 2:** Study groups’ TGF-β1, CTGF, and other laboratory parameter results

	Group 1 (n=50)	Group 2 (n=45)	P
**TGF-β1**			
1st day (ng/mL)	3.86±1.86	2.95±1.14	0.005^a^
7th day (ng/mL)	13.14±5.10		<0.001^b^
**CTGF**			
1st day (pg/mL)	762.14±465.10	276.96±146.46	<0.001
7th day (pg/mL)	2291.27±1323.42		
**WBC**			
1st day (cells/µL)	4939.80±1204.61	6892.19±1638.15	<0.001^a^
7th day(cells/µL)	6556.24±1535.87		0.305^b^
**Neutrophil**			
1st day (cells/µL)	2846.20±959.09	3953.56±995.86	<0.001^a^
7th day (cells/µL)	3702.5±1212.64		0.276^b^
**Lymphocyte**			
1st day (cells/µL)	1152±413.03	2000.56±654.63	<0.001^a^
7th day (cells/µL)	2319±2270.656		0.366^b^
**LDH**			
1st day (U/L)	427.24±213.79	192.84±39.62	<0.001^a^
7th day (U/L)	199.62±32.50		0.363^b^
**D-DIMER**			
1st day (ng/mL)	1310.74±935.06	290.31±102.84	<0.001^a^
7th day (ng/mL)	324.1±128.77		0.164^b^
**Troponin-I**			
1st day (ng/L)	5.79±7,81	2.5±1.33	0.006^a^
7th day (ng/L)	2.38±1.44		0.669^b^
**ESR**			
1st day (mm/h)	44.8±27.35	9.24±3.63	<0.001^a^
7th day (mm/h)	10.52±8.84		0.37^b^
**CRP**			
1st day (mm/h)	75.89±58.23	3.63±1.42	<0.001^a^
7th day (mm/h)	4.37±2.27		0.062^b^
**Ferritin**			
1st day (ng/mL)	453.12±312.72	208.49±66.48	<0.001^a^
7th day (ng/mL)	183.23±74.47		0.086^b^
**Procalcitonin**			
1st day (ng/mL)	0.13±0.43	0.05±0.08	0.194^a^
7th day (ng/mL)	0.03±0.02		0.189^b^

Values are presented as the mean ± standard deviation. **a:** Group 1-1st day and Group 2, **b:** Group 1-7th day and Group 2. **TGF-β1:** transforming growth factor-beta 1; **CTGF:** connective tissue growth factor; **WBC:** white blood cell; **LDH:** lactate dehydrogenase; **ESR:** erythrocyte sedimentation rate; **CRP:** C-reactive protein.

**FIGURE 1:**
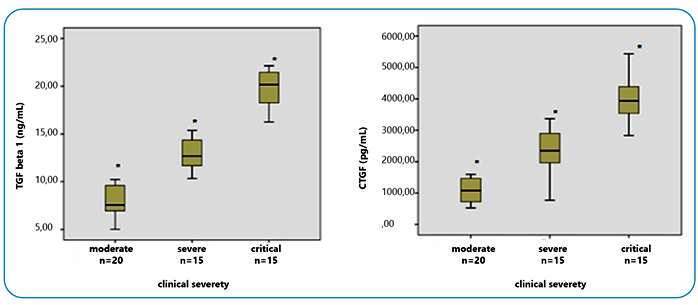
Box-plot presentation of serum TGF-β1 and CTGF levels on day 7 of admission in Group 1 divided by severity of COVID-19 disease. *:p<0.001 (analysis of variance) two-way comparison of subgroups.

TGF-β1 and CTGF levels of the patients in Group 1 were compared in terms of clinical severity (Figure 1). Serum TGF-β1 and CTGF levels correlated with clinical severity levels. The critical patient group had the highest serum TGF-β1 and CTGF levels on day 1 and 7. Two-way comparisons of all groups established on the basis of clinical severity revealed significant variation between CTGF levels on day 1 and 7. However, while there was no statistically significant difference between day 1 serum TGF-β1 levels in the two-way comparisons, a significant variation was observed on day 7 (Table 3). 

**TABLE 3:** Changes in serum TGF-β1 and CTGF levels among disease severity subgroups.

	Moderate pneumonia (n=20)	Severe pneumonia (n=15)	Critical level (n=15)	P
**TGF-β1**				
1st day (ng/mL)	3.59±1,67	3.78±1.76	4.31±2.20	>0.05*
7th day (ng/mL)	8.51±2.54	12.86±1.67	9.59±2.03	<0.001*
**CTGF**				
1st day (pg/mL)	446.13±250.30	750.88±449.03	1194.74±356.59	<0.05*
7th day (pg/mL)	1080.15±373.95	2385.23±704.44	3812.13±927.88	<0.001*

Values expressed as mean±standard deviation. *Two-way comparison of subgroups.

Serum TGF-β1 levels were moderately, positively, and significantly correlated with D-dimer, ESR, ferritin, CRP, and LDH levels (r = 0.692, r = 0.724, r = 0.545, r = 0.808, and r = 0.663, respectively; *P*< 0.001 for all). In addition, CTGF levels were positively and moderately correlated with D-dimer, ESR, ferritin, C-reactive protein, and lactate dehydrogenase levels (r = 0.671, r = 0.645, r = 0.576, r = 0.800, and r = 0.694, respectively; *P* < 0.001 for all). Serum TGF-β1 and CTGF levels were strongly correlated with CRP levels.

**FIGURE2:**
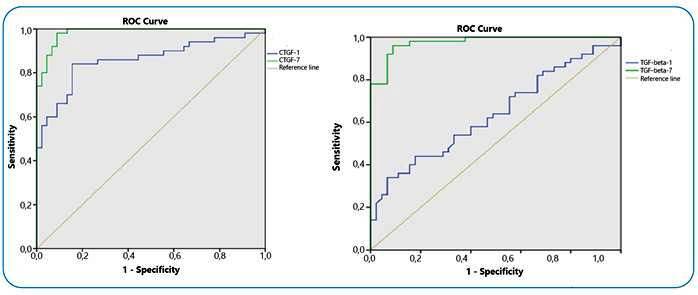
Determination of the diagnostic sensitivity and specificity of serum TGF-β1 and CTGF levels on days 1 and 7 of admission in patients diagnosed with COVID-19 using ROC curve analysis. **ROC:** receiver-operating characteristic curve.

The ROC curve method was used to establish the diagnostic sensitivity and specificity of COVID-19 patients’ serum TGF-β1 and CTGF levels. At cutoff values of 345.51 pg/mL for day 1 serum CTGF and 626.2 pg/mL for day 7 serum CTGF (AUC = 0.858, *P*< 0.001, 95% confidence interval [CI] = 0.78 - 0.94 and AUC = 0.984, *P* < 0.001, 95% confidence interval [CI] =0.85 - 0.99 respectively), sensitivity was 84% and 94%, and specificity was 82% and 91%, respectively, for distinguishing patients with diagnosed with COVID-19 with lung injury from those without lung injury (Figure 2).

At cutoff levels of 3.39 ng/mL for serum TGF-β1 on day 1 and 5.36 ng/mL for serum TGF-β1 on day 7, (AUC = 0.638, *P* = 0.021, 95% confidence interval [CI] = 0.53 -0.75 and AUC = 0.976, *P* < 0.001, 95% confidence interval [CI] = 0.82 - 0.97 respectively), sensitivity was 64% and 96%, and specificity was 51% and 89%, respectively, for discriminating patients diagnosed with COVID-19 developing lung injury from those without lung injury (Figure 2).

## DISCUSSION

The principal findings of this prospective study may be summarized as follows. First, COVID-19 patients with lung injury showed higher TGF-β1 and CTGF levels than the control group. Second, routine laboratory test values began to fall on the seventh day, after medical treatment. However, serum TGF-β1 and CTGF levels continued to increase. They may be a useful guide for clinicians in evaluating lung damage. Third, TGF-β1 and CTGF levels on day 7 in particular exhibited high sensitivity and specificity for distinguishing COVID-19 patients with lung damage from those without such damage. Finally, both TGF-β1 and CTGF levels significantly correlated with illness severity in COVID-19 patients. The study outcomes emphasize the role of TGF-β1 and CTGF in lung injury in COVID-19 patients.

TGF-β is synthesized as an inactive large precursor protein called the latency-associated peptide (LAP). LAP, a latent TGF-β is unable to bind to its receptor and is known as the inactive form of TGF-β. The intracellular protease cleaves LAP-1 from the rest of the protein to yield the biologically active TGF- β^23^. TGF-β is a multifunctional cytokine known to exhibit profibrogenic, anti-inflammatory, and immunosuppressive activities that increase during and after both sepsis and COVID-19, with the probability of halting the hyperinflammatory response^24^. TGF-β is also a powerful immunosuppressive factor that significantly inhibits immune functions and delays recovery by reducing cell recruitment and downregulating cytokine production^25^ ^,^ ^26^.

SARS-CoV-2, the agent involved in the transmission of COVID-19, causes pulmonary parenchymal damage through ACE2^27^. Damage occurring in the lung tissue leads to the overexpression and release of cytokines and a series of growth factors, such as TGF-β and CTGF^28^. Studies have shown an association between COVID-19 and ARDS, which causes widespread lung damage. Pulmonary fibrosis can develop in long-term ARDS patients^29^. Pulmonary fibrosis characteristically exhibits the overproduction and accumulation of extracellular matrix proteins, including fibronectin. COVID-19 patients with pulmonary involvement have a high risk of pulmonary fibrosis^30^. Although SARS-CoV-2 infection significantly affects lung tissue, serum TGF-β1 and CTGF levels in Covid-19 patients on day 1 and 7, have not been previously reported. The multifunctional roles of TGF-β and CTGF in lung injury and fibrosis are well known^13^ ^,^ ^15^ ^-^ ^17^.

In their study of transgenic mice, Ponticos *et al*. demonstrated the profibrotic effect of CTGF in pulmonary fibrosis and reported that CTGF may represent a target molecule in the treatment of diseases that progress with fibrosis^31^.

Cui *et al*. showed that inhibition of the NF-kB/TGF-beta/Smad2/3 pathway in mice exhibited a protective effect against pulmonary fibrosis^32^.

Increased synthesis of TGF-β and its correlation with disease severity have also been reported in chronic lung diseases, such as idiopathic pulmonary fibrosis^33^.

Xu *et al*.^34^ investigated the mechanism of pulmonary fibrosis in patients with COVID-19 pneumonia. The authors reported that SARS-COV-2 virus binds to the ACE-2 receptor, and that this activation activates genes associated with fibrosis. They also suggested that expression of proteins such as TGF-β and CTGF at the genetic level increases as a result of that activation, and that the various stages of pulmonary fibrosis are stimulated. In the present study, TGF-β1 and CTGF levels on day 1 and 7 were higher in COVID-19 patients than in the control group (*P* < 0.05). The levels of ESR, CRP, LDH, troponin-I, and D-dimer used in the diagnosis and follow-up of treatment in COVID-19 patients were higher than those in the control group on day 1 (*P* < 0.05), while WBC, neutrophil, and lymphocyte values were low (*P* < 0.05). Laboratory tests on day 7 of treatment approached the reference range in the healthy control group (*P* > 0.05). However, TGF-β1 and CTGF levels continued to increase (*P* < 0.05). This suggests that the tests used are insufficient for follow-up of the damage caused by the virus in the lungs. However, TGF-β1 and CTGF levels correlated with disease severity.

Radiography and computed tomography are of indisputable importance in the diagnosis and follow-up of lung damage in COVID-19 patients. However, notwithstanding their considerable benefits to both diagnosis and disease management, the most important disadvantage of the widespread use of imaging methods, is their potential contribution to radiation exposure and the spread of disease. Disease transmission in radiology units is primarily caused by surface contamination following droplet spread^35^. Radiological imaging revealed various lesions in the lung parenchyma of patients included in the present study. Day 7 serum TGF-β1 and CTGF levels exhibited high sensitivity and specificity for differentiating lung damage in COVID-19 patients (sensitivity, 96%; specificity, 89% for TGF-β1; sensitivity, 94%; and specificity, 91% for CTGF). There is an urgent need for new assistant laboratory markers capable of evaluating lung damage in COVID-19 patients, which can be easily analyzed and interpreted with high sensitivity and specificity, and that can be measured in blood. TGF-β1 and CTGF are two important potential candidates for use in blood tests and imaging methods. 

In conclusion, the findings of this study suggest that serum TGF-β1 and CTGF levels may be used to estimate clinical severity in patients with COVID-19 and pulmonary involvement. Further studies investigating the power of serum TGF-β1 and CTGF levels in predicting the development of persistent lung disease in patients with COVID-19 are required. Moreover, the efficiency of various anti-TGF-β1 therapies in attenuating post-COVID lung sequelae should be investigated.

## SUPPLEMENTARY MATERIAL

**SUPPLEMENTARY TABLE 1:** Frequencies and morphological characteristics of patient lesions on computed tomography.

Tomography Findings	Patient	
	Number	%
Ground glass	34	68
Consolidation	23	46
Ground glass+consolidation	21	42
Nodule	8	16
Septal thickening	10	20
Fibrosis	11	22
Air bronchogram	7	15
Reverse halo	3	6
Pleural effusion	5	10
